# From dual targeting to dynamic targeting: Rethinking CAR-NK cell therapy for pancreatic cancer

**DOI:** 10.1016/j.omton.2026.201358

**Published:** 2026-10-20

**Authors:** Kathrine S. Rallis, Laura M. Dionisio, Mubin Tarannum, Rizwan Romee, José A. Cancelas

**Affiliations:** 1 Department of Medical Oncology, Dana-Farber Cancer Institute, Boston, MA 02215, USA

## Introduction

Pancreatic ductal adenocarcinoma (PDAC) remains one of the most formidable challenges for cellular immunotherapy. Although chimeric antigen receptor (CAR)-therapies have transformed hematologic malignancies, success has not extended to solid tumors, where, physical exclusion, immunosuppression, metabolic constraints, and antigen heterogeneity limit efficacy.^1^ The dense stroma of PDAC underlies adversity. In this issue of *Molecular Therapy Oncology*, Gehrcken et al.^2^ address an important question: can simultaneous targeting of malignant cells and their supporting stroma overcome barriers and improve CAR-natural killer (NK) cell therapy for PDAC?

The authors identify mesothelin (MSLN) and CD70 as complementary targets, MSLN predominantly marking tumor and CD70 expressed on tumor and, importantly, cancer-associated fibroblasts (CAFs).^2^ NK-92-cells engineered with MSLN- or CD70-specific CARs, with or without IL-15 armoring, were evaluated across cell lines, patient-derived organoids with CAFs, and *in vivo* PDAC xenografts. Results are provocative: IL-15 enhances CAR-NK activity in several settings, whereas combining MSLN- and CD70-directed CAR-NK cells does not produce anticipated improved tumor control.

Rather than simply representing a negative result, observations expose a fundamental challenge in translating CAR-based therapies to solid tumors: adding targets does not necessarily overcome downstream barriers to antigen recognition.

## IL-15 exposes the importance of effector-cell fitness

The enhanced activity of IL-15-containing products reinforces an increasingly important principle in CAR-NK biology: successful antigen recognition is necessary but insufficient. Effector cells must expand, persist, serially kill, and remain functional within a hostile tumor microenvironment (TME). Clinical experience with cytokine-supported and cytokine-induced memory-like NK cells similarly emphasizes the importance of cellular fitness and persistence.^3^^,^^4^

Endogenous cytokines improve effector-cell-fitness and persistence.^5^ Transient mRNA-based strategies limit undesirable cytokine exposure but may be mismatched to tumors requiring durable control. CAR abundance peaks at 24 h and declines by 48–72 h, hinting at short-lived activity as limiting to observing pooled-targeting benefit. Consistently, clinically, durability limits favorable safety and activity of CAR-NK-therapies. The observed cytotoxicity, a frequent consequence of elevated IL-15 signaling, could reflect IL-15 toxicity but also increased CAR abundance. The observation that IL-15 mRNA itself increases cytotoxicity against several targets further demonstrates a CAR-independent contribution.^2^

This distinction is mechanistically important. Matching CAR surface abundance through mRNA titration, quantifying IL-15 production and STAT5 activation, and measuring proliferation, survival, persistence, and serial killing would establish whether IL-15 primarily potentiates CAR signaling or instead improves the underlying fitness of the NK-cell product. A factorial CAR-by-IL-15 design could further distinguish synergy from additive effects.

More broadly, findings suggest that the therapeutic value of cytokine armoring may lie less in increasing instantaneous cytotoxicity than in extending the functional lifetime of CAR-NK cells.

## More targets do not necessarily solve a multidimensional problem

The rationale for combined MSLN/CD70 targeting is compelling. In principle, MSLN-directed cells attack malignant cells while CD70-directed cells eliminate tumor and stromal compartments, dismantling CAF-mediated infiltration barriers. Importantly, the authors evaluate antigen expression directly in primary and metastatic human PDAC, strengthening the translational rationale for these targets. Nevertheless, antigen expression is heterogeneous, and pooled MSLN- and CD70-CAR-NK cells fail to outperform individual products across two-dimensional cultures, patient-derived organoids, and *in vivo* models. This consistency across experimental systems is informative. It challenges the intuitive assumption that expanding antigen coverage will necessarily translate into greater therapeutic activity.

The experimental design, however, is critical for interpreting results. Combination groups contained half as many cells of each CAR specificity as corresponding monotherapy groups.^2^ Consequently, these experiments tested fixed-total-dose mixture dose-efficiency versus individual products, rather than directly determining potential synergy of dual targeting. Full-dose combinations, half-dose monotherapy controls, or CAR-NK cells mixed with equivalent numbers of control NK cells would more clearly distinguish dual-target benefit from component-dose dilution.

The appropriate conclusion is therefore not that dual targeting is ineffective but that a fixed-dose mixture of transiently engineered MSLN- and CD70-CAR-NK-92 cells does not overcome PDAC resistance under conditions tested.

This distinction matters because antigen heterogeneity represents only one layer of solid-tumor resistance. Even perfect antigen coverage cannot compensate for inadequate trafficking, insufficient persistence, stromal exclusion, TGF-β signaling, metabolic suppression, extracellular matrix barriers, or tumor-intrinsic resistance.

## The model may determine the apparent limits of the strategy

The progression from conventional cultures to patient-derived organoids represents an important strength of the study. However, even sophisticated organoids reproduce only selected dimensions of PDAC biology. A single CD70-positive CAF line cannot encompass the functional diversity of inflammatory, myofibroblastic, antigen presenting, and other stromal states shaping tumor progression and immune exclusion. Patient-matched multicellular models incorporating heterogeneous stromal and immune compartments could reveal interactions invisible in simplified systems.

Choosing NK-92 cells introduces equally important biological constraints. NK-92 cells provide a reproducible engineering platform, but their receptor repertoire, cytokine responsiveness, trafficking, and persistence differ from primary, cord-blood-derived, or induced pluripotent stem cell-derived NK cells. Importantly, their requirement for irradiation limits proliferative capacity and persistence,^6^ properties that may determine whether stromal remodeling can precede and facilitate subsequent tumor-cell elimination.

The *in vivo* experiments therefore cannot fully distinguish failure of the targeting concept from limitations of the cellular platform, transient CAR expression, dose, route, trafficking, or persistence. Pharmacokinetic and pharmacodynamic measurements (including circulating and intratumoral CAR-NK persistence, retention, spatial localization, tumor-cell and CAF depletion, and dynamic changes in MSLN and CD70 expression) would sharpen mechanistic interpretation.

Clinically scalable allogeneic CAR-NK studies demonstrate feasibility of engineered NK cells.^7^ For solid tumors, engineered cell localization, retention, and functional fitness may matter as much as antigen recognition.

## From simultaneous targeting to spatially and temporally coordinated therapy

An even more powerful strategy may be to integrate multiple functions within a single engineered effector.^8^ In a phase 1/2 trial of cord-blood-derived CD19-CAR-IL-15-NK cells, response was associated with higher levels and longer persistence of infused cells, while donor-product characteristics affected outcome.^5^ iPSC-derived MSLN-CAR-IL-15-NK cells have shown enhanced tumor homing, proliferation, and activity in solid tumors.^9^

Bicistronic, tandem, logic-gated, and inducible CAR architectures could permit individual NK cells to respond dynamically to heterogeneous antigen landscapes. Chemokine-receptor engineering could improve trafficking, matrix-remodeling programs could facilitate penetration, resistance to TGF-β and metabolic stress could sustain function, and regulated cytokine circuits could support persistence while limiting systemic toxicity.

The next generation of CAR-NK therapy for solid tumors may therefore require moving beyond the concept of multi-antigen targeting toward multidimensional engineering.

## A platform-defining rather than target-defeating study

The importance of the study by Gehrcken et al. lies not simply in whether MSLN and CD70 constitute the optimal target pair. Its broader contribution is demonstrating experimentally that increasing antigen coverage alone does not necessarily translate into improved efficacy when dominant barriers reside elsewhere in the tumor ecosystem.

Several conclusions should remain appropriately bounded: the experiments do not establish uniform CAR-specific potentiation by IL-15, the general failure of dual targeting, or *in vivo* safety. Yet, limitations do not diminish the study’s significance. Instead, they sharpen its central message.

For PDAC and potentially other treatment-refractory solid tumors, successful CAR-NK therapy will likely require simultaneous optimization across multiple biological dimensions: recognition, trafficking, penetration, persistence, metabolic fitness, stromal remodeling, and resistance to immunosuppression. Antigen specificity determines what an engineered cell can recognize; cellular fitness and microenvironmental adaptation determine whether that recognition becomes durable tumor control.

Thus, failure of a pooled MSLN/CD70 strategy should not be viewed as target defeating but as platform defining. The next advance in solid-tumor CAR-NK therapy may come not from adding another target or cytokine but from engineering cells capable of sensing, surviving, navigating, and dynamically remodeling the tumor ecosystem.

## Acknowledgments

The authors wish to acknowledge grant funding from the United States NCI (R01 CA302945, J.A.C.), NIDDK (R01 DK124115, J.A.C.), NHLBI (5T32HL066987-23 and P01HL158688, K.S.R. and J.A.C., respectively), Blood Cancer United (TRP, J.A.C.), Blood Cancer United (Career Development Award, R.R.), and Parker Institute for Cancer Immunotherapy at Dana-Farber Cancer Institute. (R.R.). The funders had no role in study design, data collection and analysis, decision to publish, or preparation of the manuscript.

## Author contributions

K.S.R., conceptualization, drafting, figures, and revisions; L.M.D., drafting and revisions; M.T., revisions; R.R., revisions and supervision; J.A.C., conceptualization, drafting, revisions, and supervision.

## Declaration of interests

R.R. is a scientific co-founder of InnDura Therapeutics and serves on the scientific advisory board of Glycostem Therapeutics.
